# Isolation and Identification of *Ipomoea cairica* (L.) Sweet Gene *IcSRO1* Encoding a SIMILAR TO RCD-ONE Protein, Which Improves Salt and Drought Tolerance in Transgenic *Arabidopsis*

**DOI:** 10.3390/ijms21031017

**Published:** 2020-02-04

**Authors:** Benqi Yuan, Minghao Chen, Shaoshan Li

**Affiliations:** Key Laboratory of Ecology and Environmental Science in Guangdong Higher Education, School of Life Sciences, South China Normal University, Guangzhou 510631, China; yuanbenqi@m.scnu.edu.cn (B.Y.); 2017022212@m.scnu.edu.cn (M.C.)

**Keywords:** *Ipomoea cairica*, SIMILAR TO RCD-ONE protein, gene expression, transgenic Arabidopsis, salttolerance, drought, invasive plant

## Abstract

*Ipomoea cairica* is a tropical plant and a wild relative of the food plant sweet potato (*Ipomoea batatas*), listed as one of the most invasive alien species in China. Recently, it has been reported that *I. cairica* had successfully invaded mangrove wetlands, indicating its high salt tolerance. Based on previous genetic studies, *I. cairica* offers a good model for characterizing stress-resistant genes. It has recently been identified that the SRO proteins (SIMILAR TO RCD-ONE) play important roles in a variety of stress and developmental responses. Radical-Induced Cell Death1 (RCD1) was the first identified plant SRO protein from *Arabidopsis thaliana*. As a typical SRO protein, IcSRO1 had a highly conservative WWE domain, a conserved PARP fold and protein C in the RST function area. The expression of IcSRO1 was induced by salt, drought, and the plant hormone ABA. The transgenic Arabidopsis overexpressing IcSRO1 showed higher tolerance against salt and drought stress along with lower accumulation of hydrogen peroxide (H_2_O_2_) and superoxide (O_2_^−^) than the wild type. The IcSRO1 protein was localized in the nucleus after cultivation in the buffer. Our results indicated it could interact with *Arabidopsis* SALT OVERLY SENSITIVE 1 (AtSOS1), suggesting IcSRO1 may have similar functions. The pleiotropic effect of IcSRO1 on physiological processes contributes to the improvement of plant tolerance against diverse abiotic stresses, and may be associated with the adaptation of *I. cairica* to those environments with extreme saline and drought conditions. It therefore provides valuable gene resources for crop breeding enhancement.

## 1. Introduction

*Ipomoea cairica* (L.), Sweet (Convolvulaceae), is an extremely fast-growing perennial creeping liana, believed to be native from tropical Africa or South America [1,2,3,4]. In China, *I. cairica* started its invasion of Hong Kong in 1912, and now it has expanded widely to most of the provinces in South China [5]. This vine has invaded natural landscapes, abandoned farmland, roadsides, residential areas, and even artificial forests and has caused severe damage to ecosystems and local economies [6]. It has been listed as one of the worst invasive alien species in China [7]. It exhibits high nutrient uptake and utilization efficiency, greater resistance to many diseases and strong adaptive abilities to diverse habitats [8]. Recently, it was shown that *I. cairica* is resistant to salt. However, it has cross-incompatibility with closely related species in the genus *Ipomoea* [9]. As an important food, industrial, and energy resource crop, there is a dramatically increased demand for *Ipomoea batatas* (L.) Lam (sweet potato), but its yield is often restricted by various biotic and abiotic stresses. Based on previous genetic studies, *I. cairica* offers a good genetic model species for characterizing stress-resistant genes. Application of these genes in transgenic breeding is expected to improve the resistance of crops, which is also the potential value of *I. cairica*. [10].

Abiotic stresses are significant limiting factors that affect global crop production, including salinity and drought. At present, salinity has become a major problem suffered by 50% of the irrigated lands and over 30% of cultivated lands all around the world [11]. The increased salinization of arable land will bring about a loss of 30% agriculture lands within two decades [12]. In China, over 1.0 × 10^8^ hm^2^ of cultivated lands suffers from saline soils, occupying approximately 7% of the total [13]. Arid and semi-arid land also accounts for one-third of the Earth’s land, and the rest has a high potential to face unexpected climatic droughts from time to time [14]. Salt and drought stress leads to severe production reduction in agriculture, and it may be feasible to meet the challenge of salinity and drought by improving the resist ability of crops using plant genetic engineering [15,16,17,18,19].

The function of SRO proteins, also named as SIMILAR TO RCD-ONE, participating in a variety of stress and developmental responses, has been reported recently. They are hallmarked by the domain with a conserved poly (ADP-ribose) polymerase (PARP) fold and a C-terminal RST (RCD-SRO-TAF4) domain [20]. Moreover, it is known that some SRO proteins hold a WWE domain in their N-terminal sequences [20,21]. Many SROs have been further investigated in various plants, including Arabidopsis [22], rice [23], wheat [24], maize [25], tomato [26], cotton [27], *Brassica napus* [28] and apple [29]. The first SRO protein identified from *Arabidopsis thaliana* is RCD1, named after its function in cell death induced by radicals. It is a stress-associated hub, reported to interact with over 30 proteins, 21 of which are recognized as transcription factors from different families [30,31]. The mutation of *AtRCD1* results in significantly increased sensitivity to extracellular reactive oxygen species (ROS) and suppressed resistance to methyl viologen, Ultraviolet-B irradiation, salt sensitivity, heavy metals toxicity and altered responses to hormones, as well as defects in developmental processes, such as defects in root architecture, alterations to leaf and rosette morphology, and early flowering [32,33,34,35,36,37]. The interaction between RCD1 and salt overly sensitive1 (SOS1) was detected in Arabidopsis under salinity and oxide. There is a possibility that RCD1 regulates the activity of Na+/H+ antiporter of AtSOS1 and influence the salt tolerance [38].

In this present study, we describe the cloning and characterization of a homoeologous *RCD1* locus (*IcSRO1*) from *Ipomoea cairica*. Quantitative real-time PCR was used to assess the expression features of IcSRO1 under different stress treatments. Then the gene was transformed into Arabidopsis thaliana for further functional identification.

## 2. Results

### 2.1. Isolation and Bioinformatics Analysis of IcSRO1 Gene

**Figure 1:**
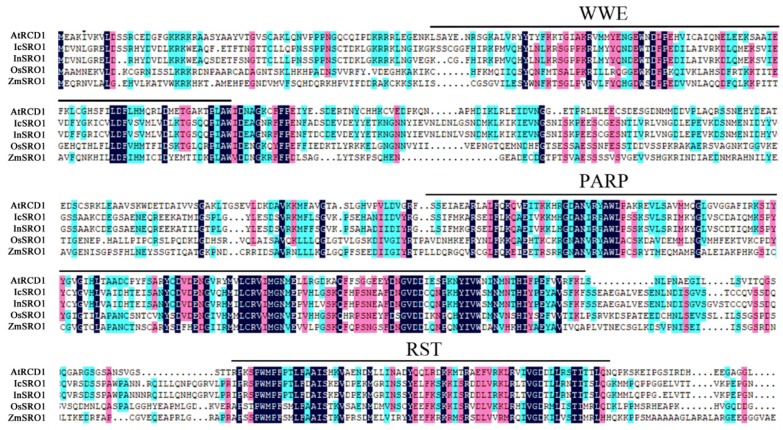
Sequence alignment of IcSRO1 with RCD1 from *Arabidopsis thaliana* and its homologies in *Ipomoea batatas*, *Oryza sativa* and *Zeamays* indicates that the amino acid sequence of IcSRO1 is similar to other species. N-terminal WWE domain, the core of the poly (ADP-ribose) polymerase (PARP), C-terminal RCD1-SRO-TAF4 domain (RST domain) are marked. Amino acid sequences of other genes were derived from NCBI (http://www.ncbi.nlm.nih.gov/).

Isolation of IcSRO1 and sequence analysis IcSRO1 was from cloned *I. cairica* and the open reading frame (ORF) comprising 1806 bp was obtained by PCR, encoding a protein of 601 amino acid residues. The protein has an isoelectric point (PI) of 5.56, and its predicted molecular weight (MW) is 67.72 kDa. We further analyzed the conserved sequence of this protein and confirmed that the IcSRO1 contained a conserved WWE domain at the N-terminal of the proteins, a conserved PARP fold and a protein C-terminal RST functional domain (Figure 1). The sequence analysis revealed that it encoded a protein homologous to a series of the SIMILAR TO RCD-ONE1 (SRO) family.

**Figure 2:**
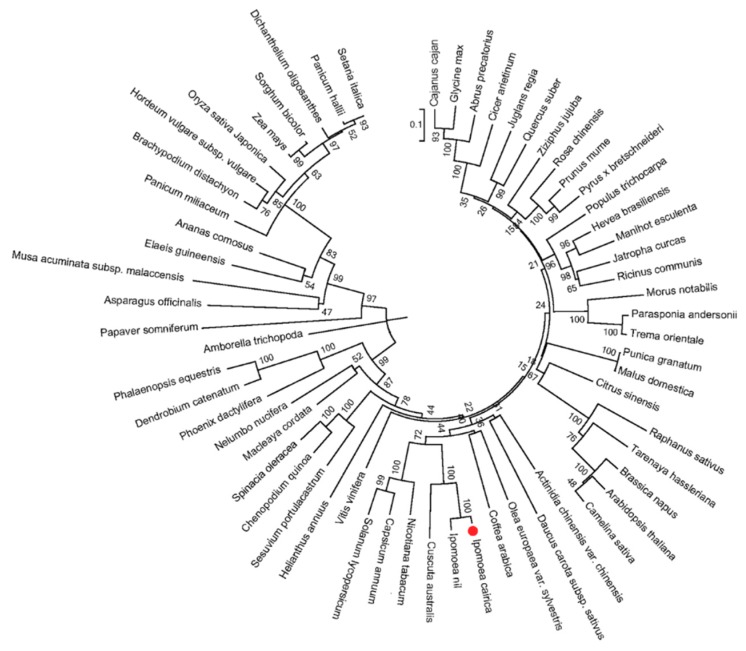
Phylogenetic tree of IcSRO1 and other SRO proteins revealed that IcSRO1 had a close relationship with the predicted protein products of *Ipomoea nil*. The tree was constructed by MEGA 6.0 software, based on alignment of complete protein sequences. The red dot indicates IcSRO1 protein.

**Figure 3:**
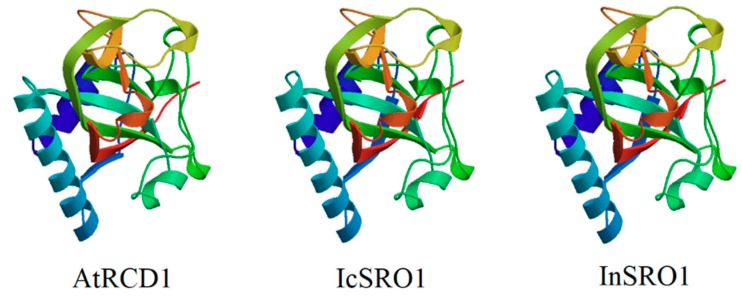
The 3D structure models show AtRCD1, IcSRO1 and InSRO1 share a similar structure. The model was constructed by SWISS-MODEL.

The amino acid sequence of IcSRO1 indicates that it is similar to other plant SIMILAR TO RCD-ONE1 proteins (Figure 1) such as *Ipomoea nil* (XP_019156087.1, 93.40%), *Cuscutaaustralis* (RAL45313.1 60.73%), *Capsicum annuum* (XP_016550729.1 49.17%), *Nicotiana tabacum* (XP_016511418.1, 50.66%), *Solanum lycopersicum* (XP_004244408.1, 48.93%), *Olea europaea* var. sylvestris (XP_022878575.1 45.08%), *Glycine max* (XP_003516978.1, 42.12%) and *Arabidopsis thaliana* (NP_564391.1, 37.40%). IcSRO1 showed high homology with morning glory (*Pharbitis nil* (L.) *Choisy.*), tobacco (*Nicotiana tabacum* L.), tomato (*Solanum lycopersicum*), and Arabidopsis. These statistics indicated that IcSRO1 has typical features of SRO-like proteins and a close relationship with some homologues in dicots. Phylogenetic analysis revealed that IcSRO1 was relative to the predicted protein products of *Ipomoea nil* (Figure 2). IcSRO1 and InSRO1 were modeled onto the RCD1 PARP-like structure (PDB 5NGO) using SWISS-MODEL [39]. The three-dimensional (3D) structure shows that IcSRO1, AtRCD1, and InSRO1 have some similarity in their structures (Figure 3).

### 2.2. Subcellular Localization of the IcSRO1 Protein

**Figure 4:**
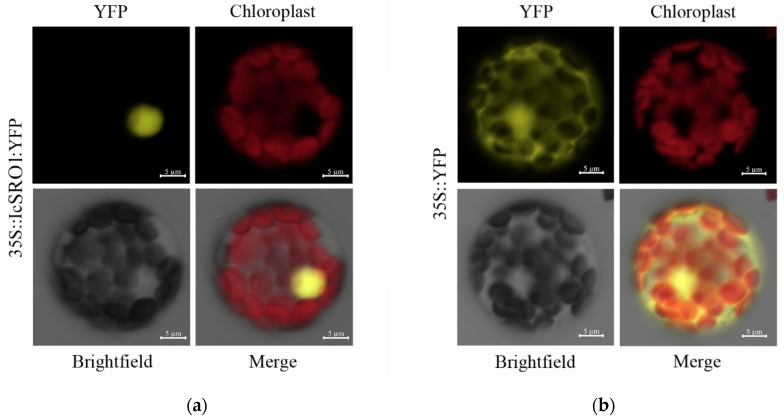
Subcellular localization of IcSRO1 protein. IcSRO1-YFP fusion protein was localized in nucleus of Arabidopsis mesophyll protoplast (**a**), while YFP was distributed throughout the entire protoplast cell (**b**). The yellow indicates yellow fluorescent, and the red represents chloroplast autofluorescence. Scale bars = 5 μm.

IcSRO1-YFP fusion protein was investigated to examine the exact subcellular localization of the IcSRO1 protein. C-terminal yellow fluorescent protein (YFP) of IcSRO1-YFP was used as a marker. The protein with a single YFP was used as a control. These constructs were transformed into Arabidopsis mesophyll protoplasts. The fluorescence was detected by confocal laser-scanning microscope. The results showed that fluorescence was mainly distributed in the nucleus of the cells expressing *IcSRO1-YFP* fusion gene; however, the fluorescence in the cells expressing the YFP was distributed throughout the entire protoplast cell, indicating that the IcSRO1 protein was localized in the nucleus (Figure 4).

### 2.3. Expression Pattern of IcSRO1 Gene under Different Abiotic Stresses

**Figure 5:**
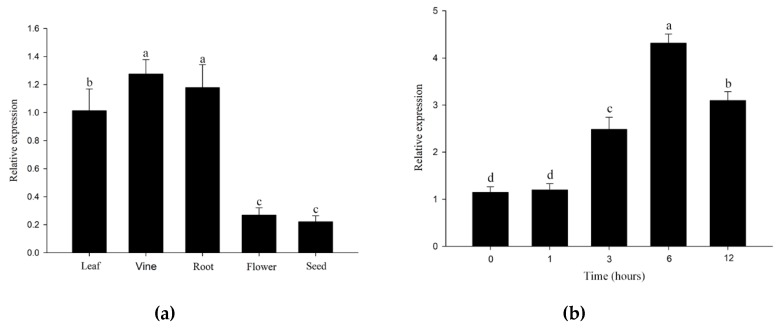

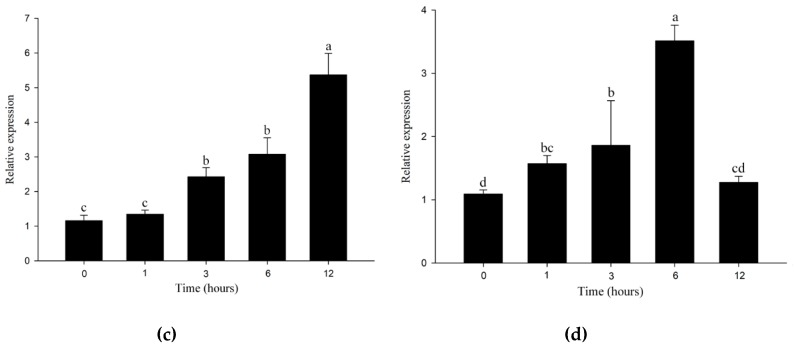
Expression profiles shows IcSRhO1 gene expresses differently in different tissues including leaf, vine, root, flower and seed (**a**). The expression of IcSRO1 is induced by different abiotic stressed: (**b**) Salt, (**c**) PEG, (**d**) ABA. The abundance of IcSRO1 transcripts increased to a peak at 6 h after salt and ABA treatments and decreased at 12 h, while they increased to the peak at 12 h after PEG treatments. The data represent the means ± SDs of three independent biological replicates. The different letters in the bar graphs indicate significant differences at *p* < 0.05.

To investigate the response of *IcSRO1* to abiotic stresses, qRT-PCR was used to analyze the relative expression levels. The abundance of *IcSRO1* transcripts changed very little in the first hour after sodium chloride (NaCl) treatment, while subsequently appeared a dramatical increasing by approximately 3.6 folds after 6 h, then followed by a decrease. The drought stress was stimulated by 20% polyethylene glycol (PEG). After drought, the *IcSRO1* expression level increased gradually to peak at 12 h. Similarly, *IcSRO1* expression was considerably elevated by exogenous 10 µM abscisic acid (ABA)(Figure 5), which can inhibit seedling and growth. In contrast, the expression of *IcSRO1* under control condition showed no difference during the entire treatment. These results manifest that *IcSRO1* responses to various abiotic stresses in the short term.

### 2.4. Overexpression of IcSRO1 Gene in Arabidopsis Enhances Resistance to Salt, Drought and Reduced ABA Sensitivity

**Figure 6:**
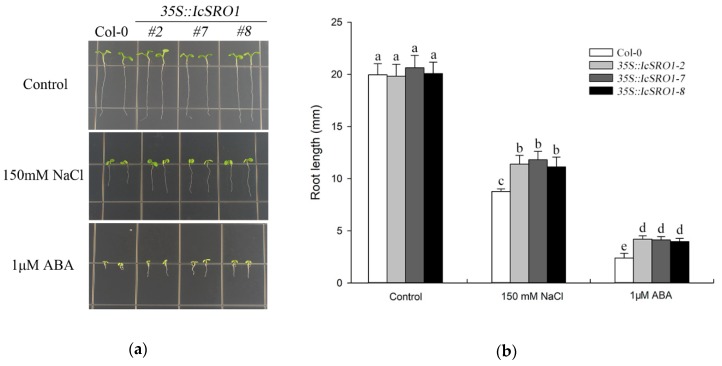

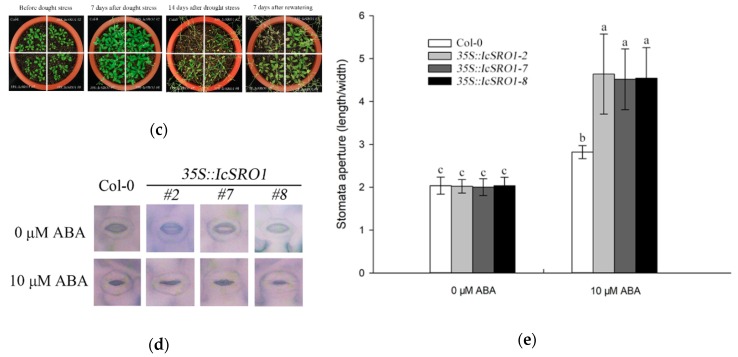
Overexpressed IcSRO1 in transgenic Arabidopsis improves plant adaptation to salinity and drought stress and reduced the sensitivity to exogenous ABA. (**a**) The transgenic Arabidopsis and Col-0 seedings were sown on medium containing 0, 150 mM NaCl, and 1μM ABA. (**b**) Length of primary roots was calculated in the transgenic and Col-0 seedings. (**c**) Drought treatments along with rewetting were applied to 2-week-old transgenic and Col-0 plants. (**d**,**e**) Stomatal aperture measurement of Col-0 and IcSRO1 transgenic plants in response to 0 and 10 μmol/L ABA. All the data represent the means ± SDs. The different letters in the bar graphs indicate significant differences at *p* < 0.05.

In order to determine the role that IcSRO1 plays in abiotic stresses, transgenic Arabidopsis plants overexpressing *IcSRO1* controlled by the CaMV35S promoter were generated. Afterwards, three independent homozygous IcSRO1 T3 lines (*35S::IcSRO1-2, 7, 8*) were selected for further functional analysis. PCR amplification was used to detect the carrying of IcSRO1 in transgenic plants and Col-0. PCR product at the length of 1800 bp was obtained in three transgenic lines, while none in Col-0. Under normal conditions, seedling growth of transgenic plants shows no evident difference from Col-0. In the other hand, the root lengths of the *IcSRO1* overexpressing lines were significantly longer than the Col-0 plants on Murashige & Skoog (MS) medium contained 150 mM NaCl (Figure 6), indicating that *IcSRO1* transgenic lines had a stronger capacity to resist salt stress.

In order to take a further step to evaluate the significance of *IcSRO1* during plant adaptation to drought, 2 weeks of water shortage was applied to transgenic and Col-0 plants which were previously grown under normal condition for 2 weeks. As a result, the drought treatment wilted the most of the Col-0 plants but a few transgenic ones by severe dehydration. The subsequent rewetting restored the *IcSRO1* overexpressing lines efficiently, with the survival rates of the transgenic lines (*35S::IcSRO1-2, 7, 8*) conspicuously higher than that of the Col-0 plants (Figure 6). These results showed that *IcSRO1* has a remarkable effect on the resistance of transgenic Arabidopsis against arid condition.

ABA signaling involves in plant responses to multiple abiotic stress. [40]. To achieve a deeper understanding how the *IcSRO1* gene functions in response to ABA, corresponding treatments with exogenous ABA were applied to the transgenic and Col-0 plants during the germination and post-germination growth periods. After exposure to ABA, root length of three transgenic lines showed is significantly longer compared to the Col-0 control (Figure 6), which reveals that exogenous expression of *IcSRO1* gene reduced the sensitivity of Arabidopsis to exogenous ABA.

It is agreed that stomatal closure during arid condition could be regulated by ABA [41]. Therefore, as *IcSRO1* expression was influenced by ABA, we compared the ABA sensitivity of stomatal closure in *IcSRO1* overexpressing transgenic plants with Col-0 controls. When exposed to 10 µmol/L ABA, the stomatal aperture of transgenic lines was smaller than that of Col-0 plants (Figure 6).

### 2.5. Determination of H_2_O_2_ and O_2_^−^Accumulation

**Figure 7:**
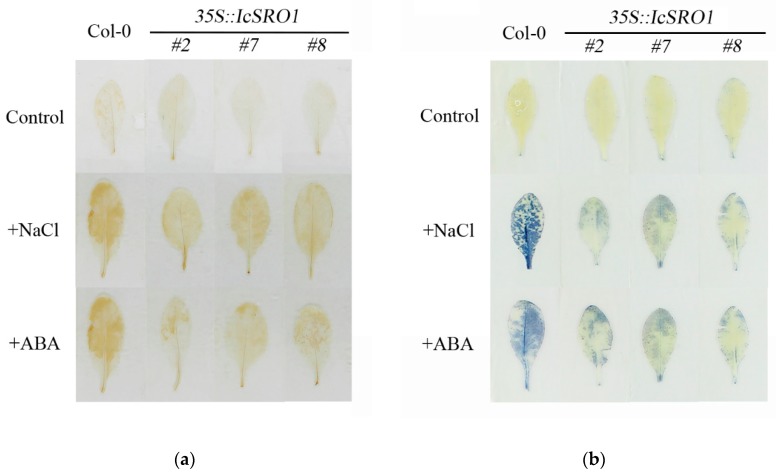
Accumulation of H_2_O_2_ and O_2_^−^ under stress conditions demonstrates more H_2_O_2_ and O_2_^−^ produced in the Col-0 than in the transgenic lines under salinity and drought stress. Histochemical staining assays were used to detect H_2_O_2_ and O_2_^−^ by DAB (**a**) or NBT (**b**) staining, respectively.

After salt and drought stress, leaves of transgenic and non-transgenic Arabidopsis were stained by 3,3-diaminobenzidine (DAB) and nitroblue tetrazolium (NBT) to detect the production and distribution of H_2_O_2_ and O_2_^−^. Under the optimum condition, no difference shows up between the transgenic and non-transgenic. Stained by DAB, leaves of col-0 appeared obvious dark-brown color under stress conditions compared to non-stress condition, while leaves of transgenic lines appeared light-brown color under stress conditions. Stained by NBT, leaves of col-0 showed dark-blue under stress conditions compared to non-stress condition, while leaves of transgenic lines showed light-blue under stress conditions (Figure 7). The results demonstrate more H_2_O_2_ and O_2_^−^ accumulation in the non-transgenic plants under salinity and drought stress.

### 2.6. IcSRO1 Protein Interacts with AtSOS1 Protein

**Figure 8:**
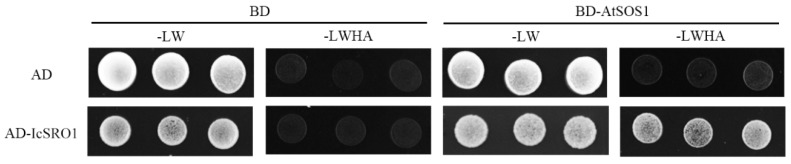
Interaction of IcSRO1 with AtSOS1 determined by yeast cell growth on synthetic dropout (SD) medium lacking Leu, Trp, His, and Ade (SD-LW HA).

Previous studies have shown that AtRCD1 interacted with AtSOS1. In this paper, the interaction between the IcSRO1 protein and AtSOS1 was investigated via Yeast Two-hybrid Assays. The ORF of IcSRO1 was inserted into pGADT7. The trans-membrane domain of AtSOS1 in its N-terminal region was excluded, and the intracellular domain was cloned into the pGBKT7 vector. As shown in Figure 8, all of the yeast transformants grow normally on the double dropout medium while only the (AD-IcSRO1+BD-AtSOS1) yeast transformants survived on the quadruple dropout medium. The results showed that the IcSRO1 protein could interact with the intracellular domain of AtSOS1 protein.

## 3. Discussion

*Ipomoea cairica*, a member of the Ipomoea, distributed in the tropics and have a close relationship with sweet potato. Soil salinity and drought constitute a major factor limiting crop production affecting plant growth and survival. The SRO proteins family remains highly conservative during evolution and is found in many plant species including Arabidopsis, rice, wheat, maize and apple. Previous reports identified several SROs and how they function in different kinds of basic cellular processes. These proteins involve in plant adaptation to stress and growth development [20,34,42,43,44]. Nevertheless, the functions of SROs still remain unknown in perennial creep lianas, especially in members of Ipomoea.

In this paper, we firstly reported the isolation of a new SRO protein from *Ipomoea cairica*. Sequence alignment along with phylogenetic analysis involving several species demonstrated that the IcSRO1 gene is a putative *Ipomoea nil* ortholog of the InSRO1 proteins and thus is named IcSRO1. As a typical RADICAL-INDUCED CELL DEATH1 protein, IcSRO1 remains a highly conservative structure similar to its homologies, including a WWE domain, reported as a conserved PARP fold and protein C in the RST function area. In our research the function of IcSRO1 was validated, contributing to a better understanding of the similarities and differences among SRO genes across diverse species. The expression levels of the *IcSRO1* gene distinguish in different tissues of *I. cairica*. It was found that the expression levels are significantly higher in leaves and vine than in root, flower and seed. The expression of IcSRO1 was induced by salt, drought, and ABA, which was similar to that of Arabidopsis by modulating ROS homeostasis [34,36,38]. Our results are consistent with previous reports [45,46]. In maize, expression of SRO genes was detected in different tissues and plant development stages. Expression of *ZmSROs* was similarly regulated by salt, drought, ABA, and gibberellic acid (GA) as well as *MdRCD1* In *Malus domestica* [29]. *OsSRO1* was reported as a target gene of *SNAC1*, which enhances plant sensitivity to abscisic acid. *OsSRO1* declines the opening level of stomata and thus lowering transpiration mediated water loss [30,47,48]. In Arabidopsis, DREB2A was reported as a transcription factor inducing expression of downstream genes related to salt and drought resistance and contributed to plant stress-defense [49]. AtDREB2A is located on the ABA-independent signaling pathway [50]. Vainonen et al. found that the AtDREB2A protein stability could be post-translationally regulated by AtRCD1 and the *rcd1* mutant is not generally ABA insensitive which indicates the RCD1–DREB2A is probably not acting in ABA signaling [44]. It is implied that IcSRO1 may share the similarity.

During the past several decades, analyses of the functions, structures and other characteristics of genes, were mainly based on model plants like Arabidopsis, Rice and Medicago. To take a deeper look into the function of IcSRO1 under abiotic stress conditions, we overexpressed *IcSRO1* in transgenic *Arabidopsis Thaliana*. Validations of phenotype and physiology indicated that overexpressed IcSRO1 remarkably improves salt and drought tolerance in transgenic plants. Overexpression *Triticum aestivum TaSRO1* in Arabidopsis can also improve salt and drought tolerance and simultaneously change the contents of catalase (CAT), ascorbate peroxidase (APX), and glutathione peroxidase (GPX) as well as survival rates [24]. Plants respond to various stresses from external environment by activating enormous and complex signaling systems. Moreover, mutual regulations and cooperation between proteins play a considerable part in stress resistance of plants. Previous studies found that as a cellular hub, AtRCD1 interacts with many transcription factors, such as AtWRKY47, AtbHLH11, and AtSTO, which have been shown to be involved in stress responses [31,47,51]. In addition to transcription factors, AtSOS1, the plasma membrane localization antiporter, interacts through its C-terminal predicted cytosolic tail with AtRCD1. Under non-stress condition, IcSRO1 was localized in nucleus. However, it has also been found that the AtRCD1 is transported out of the nucleus under salt stress conditions suggesting that AtRCD1 plays a critical role in the cross-talk between the ion-homeostasis and oxidative-stress detoxification pathways involved in plant salt tolerance [38]. In our research, IcSRO1 could also interact with intracellular domain of AtSOS1, which suggests that IcSRO1 may have the similar functions. However, the subcellular localization is performed in mesophyll protoplasts, with potential limited gene expression due to heterochromatins and physiological changes caused by cell wall digestion. The gene localization might be influenced by these factors and should be verified further.

In summary, our findings indicated that IcSRO1 shares close homology with AtRCD1 and other SROs. IcSRO1 was localized in nucleus and induced by salt drought and ABA, and interacts with the intracellular domain of AtSOS1. The overexpression of IcSRO1 enhances the adaptation of plants to salt and drought stress. This IcSRO1-involved adaptation may promote the invasion of *I. cairica* in tropical saline-alkali lands and coastal zones. There is a potential value of *I. cairica* for improving the resistance of sweet potato against environmental stress due to their relationship, which has profound significance for improving breeding programs aimed at improving salt and drought resistance.

## 4. Materials and Methods 

### 4.1. Plant Material and Growth Conditions

The *I. cairica* seeds were collected from the intertidal zone of Qi’ao Island (GPS coordinate: 22.44° N, 113.63° E), Zhuhai city, Guangdong province, China. For *I. cairica* seedling culture, the seeds were sterilized for 5 min with commercial bleach and washed in sterile water. The seed coats were removed with caution by sandpaper. Coat-removed seeds were placed in Petri plates with two layers of moist filter paper in it, and then transferred to a climate control cabinet (MGC-850HP, Yiheng Technology Co., Ltd., Shanghai, China) for cultivation. The cultivation condition was 25 °C, 14h light/10 h dark photoperiod, and 60% humidity. Seedlings were transferred to half-strength Hoagland’s nutrient solution in small black containers under the same growth conditions [52]. The nutrient solution was refreshed every two days.

### 4.2. Isolation and Bioinformatics Analysis of IcSRO1

100 mg of tissue was taken from each plant and frozen in liquid nitrogen. Total RNA was carefully extracted by TRIzol reagent (Takara, Japan). Absorbance values of the extract at 230 nm, 260 nm and 280 nm were measured by an Agilent 2100 Bioanalyzer (Agilent Technologies, USA) to check the concentration and purity of the RNA. Gel electrophoresis was used to detect the degradation of RNA. First-strand cDNA was synthesized by PrimeScript^TM^ RT Reagent Kit with gDNA Eraser (Takara, Japan).

The full-length ORF of *IcSRO1* gene was amplified from *I. cairica* using a PCR method. The cloning primers of the predicted full-length *IcSRO1* gene were designed by Primer Premier 6.1 (Primer Biosoft, San Francisco, CA, USA) (Table S1). The gene was amplified using following PCR program: an initial denaturation at 94 °C for 1 min, followed by 30 cycles at 98 °C for 10 s, 58 °C for 30 s, 72 °C for 1 min and a final extension at 72 °C for 3 min. The amplification products were recycled and then ligated to the pMD-18 vector. The ligation products were subsequently transformed into competent *Escherichia coli* (*E. coli*) DH5α. Positive colonies were selected for culture and subjected to PCR detection. The detected positive clones were sequenced (Beijing Genomics Institute, Beijing, China).

The amino acid sequences of IcSRO1 with other RCD1 homologs from different plant species were aligned by the DNAMAN Version 9 (Lynnon Biosoft, San Ramon, CA, USA). Protein physicochemical properties were predicted by online tool Protparam of ExPASy Proteomics Server (http://www.expasy.ch/tools/protparam.html). The neighbor-joining phylogenetic tree was constructed by MEGA 6.0. Bootstrap values were estimated (with 1000 replicates) to assess the relative support for each branch [53]. The three-dimensional structural models of proteins were predicted using SWISS-MODEL Server (https://swissmodel. expasy.org/). The theoretical pI and MW of the IcSRO1 protein were predicted via the online computer program pI/MW (http://web.expasy.org/compute_pi/).

### 4.3. Subcellular Localization Analysis of IcSRO1

The open reading frame of the *IcSRO1* gene without the termination codon was amplified with specific primers (Table S1) and inserted into the pCambia-1300 expression vector containing the yellow fluorescent protein gene (*YFP*) under the control of CaMV35S promoter and NOS terminator. The empty vector containing only *YFP* sequence was used as the control. Leaves of one-week-old seedlings were harvested and the protoplast transformation was conducted according to the method reported by Yoo et al. [54]. After the transformation, the protoplasts were incubated at 23 °C in an incubator. After 12 h of incubation, a Zeiss LSM 800 confocal laser (Carl Zeiss, Jena, Germany) scanning microscope was used to observe the subcellular localization of YFP fusion protein. The excitation wavelength was 514 nm and the emission wavelength was 530–600 nm. The chlorophyll auto-fluorescence was recorded simultaneously.

### 4.4. Expression Analysis of IcSRO1 Gene in I. cairica

In order to mimic salinity and drought, and ABA stress treatments, the four-leaf stage seedlings of *I. cairica* were transferred into half-strength Hoagland’s nutrient solutions containing 150 mM NaCl, 20% PEG 6000 or 10 μM ABA. Leaves were collected at 0, 1, 3, 6, 12h after various treatments to assess the expression patterns of *IcSRO1*. The time point of 0h was set as control. *I. cairica* ICs were harvested after these treatments. Three biological replicates were taken for each treatment, then quickly placed in liquid nitrogen and stored at −80 °C for RNA extraction. The method of RNA extraction was as described previously [55].

Quantitative real-time PCR (qRT-PCR) was completed to assess the expression of *IcSRO1* in several stress treatment. qRT-PCR was conducted with the Luna^®^ Universal qPCR Master Mix (New English Biolabs Inc., USA). The operating procedure by Applied Biosystems QuantStudioTM 6 Flex Real-Time PCR system (Applied Biosystems, USA) for qRT-PCR was as follows: Initial denaturation at 95 °C for 60 s, followed by 40 cycles (15 s at 95 °C, 30 s at 58 °C), with a melting curve analysis (60–95 °C at a heating rate of 0.1 °C per second) to verify the specificity of amplicons. Three technical repetitions were set for each sample. The fluorescence level of each sample observed by qRT-PCR, known as CT values, was normalized by taking the CT value of *IcACTIN* as a reference. The comparative 2^−ΔΔCt^ method was used to calculate the relative expression level of the *IcSRO1* in each sample [56].

### 4.5. Generation of Transgenic Arabidopsis

The full-length cDNA of *IcSRO1* was amplified by PCR and cloned into the pCambia-1300 under the control of the CaMV35S promoter. The recombinant plasmid was sequenced to confirm that the target gene was inserted correctly. Afterwards, the construct was transferred into *Agrobacterium tumefaciens* GV3101 as described in the instruction manual of In-Fusion ^®^ HD Cloning Kit (TaKara, Kusatsu, Japan). Arabidopsis thaliana was infected with recombinant *Agrobacterium tumefaciens* by floral-dip method, along with the transformation of target gene. Seeds were harvested after seeding and seeded on sterilized MS medium containing 50 mg kanamycin. After 7 days of cultivation, resistant seedlings were transplanted into Arabidopsis soil and cultured for another 30 days. Validated by PCR, three homozygous transgenic lines with different expression levels were selected for further study.

### 4.6. Stress Tolerance Assays with IcSRO1 Overexpressing Transgenic Arabidopsis Plants

For stress testing of transgenic Arabidopsis overexpressing IcSRO1, Arabidopsis ecotype Columbia (Col-0) was used as the wild-type. In vitro assay for salt tolerance and ABA was conducted as described previously [29]. Transgenic Arabidopsis T3 and Col-0 seeds were sown on MS medium with 150 mM NaCl and 1 µM ABA for one week at 22 °C under 16 h of light, and then their root length was measured.

### 4.7. Histochemical Detection of Oxidation Resistance

The transgenic and Col-0 seedlings, grown on the medium for one week in advance, were transplanted into pots and cultured for another 3 weeks. Drought or salt stress treatments were carried out at the forth week. Plants were droughted by water shortage treatment for two weeks. In salt treatment the plants were subjected to salt stress by watering 150 mM NaCl. Leaves were sampled and stained by NBT and DAB (Sigma-Aldrich, St. Louis, MI, USA) as previously described by Lee et al. with a few modifications [57]. 50 mM PBS with pH 6.4 was used for NBT staining and 50 mM PBS with pH 7.0 was used for DAB staining.

### 4.8. Yeast Two-hybrid Assay

Vectors pGADT7 and pGBKT7 were used in the yeast two-hybrid assay. The full length of IcSRO1 and intracellular domain of AtSOS1 were constructed on these two vectors respectively. The pGADT7-IcSRO1 and pGBKT7-AtSOS1 vectors were transformed into competent yeast cells and cultured on double dropout medium (SD/−Trp/−Leu) and quadruple dropout medium (SD/−Leu/−Trp/−His/−Ade), respectively. The information of primers used in this study is listed in Table S1.

### 4.9. Statistical Analysis

All the experiments in this study were repeated three times independently and values were shown as the mean ± SD (*n* = 3). Data were analyzed by one-way analysis of variance (ANOVA) or two-way ANOVA. The subsequent multiple comparisons were examined based on the least significant difference (LSD) test. *p*< 0.05 was considered a significant level. Data analysis was performed with SPSS 21.0 (SPSS Inc., USA). Figures were prepared using Sigmaplot 12.5 (Systat Software Inc., San Jose, CA, USA).

## Supplementary Materials

Supplementary materials can be found at https://www.mdpi.com/1422-0067/21/3/1017/s1.
