# Supplementary material for: Isolation and Identification of Ipomoea cairica (L.) Sweet Gene IcSRO1 Encoding a SIMILAR TO RCD-ONE Protein, Which Improves Salt and Drought Tolerance in Transgenic Arabidopsis
Source: Int J Mol Sci. 2020 Feb 4;21(3):1017. doi: 10.3390/ijms21031017 (PMC7036886; doi:10.3390/ijms21031017)
Supplement: Supplementary file 1 [file ijms-21-01017-s001.pdf]

Table S1. List of primer pairs used in this study

Experiment	Name	Primer sequence (5'-3')
Clone for ORF	orfRCD1F	ATGGATGTAAACTTAGGAAGGG
	orfRCD1R	TCAGTTTCCGGGCTCTGGTTTC
Subcellular localization	subRCD1F	GGGCATATGATGGATGTAAACTTAGGAAGGG
	subRCD1R	GGGGAATTCTCAGTTTCCGGGCTCTGGTTTC
pGADT7-IcRCD1	ADRCD1F	GGGCATATGATGGATGTAAACTTAGGAAGGG
	ADRCD1R	GGGGAATTCTCAGTTTCCGGGCTCTGGTTTC
pGBKT7- AtSOS1	BDSOS1F	GAGGCCGAATTCCCGGGGATGACGACTGTAATCGAC
	BDSOS1R	TGCAGGTCGACGGATCCC TCATAGATCGTTCCTGA
Overexpression in Arabidopsis	overRCD1F	GGGCATATGATGGATGTAAACTTAGGAAGGG
	overRCD1R	GGGGAATTCTCAGTTTCCGGGCTCTGGTTTC
Real time RT-PCR	qACTINF	GCGGATAGAATGAGCAAGGAA
	qACTINR	GGGCCGGACTCATCATACTC
Real time RT-PCR	qRCD1F	CCCATAATGACTCGGCACA
	qRCD1R	TCTGAAGCAGGCTGAAATCG
